# The milk-derived fusion peptide, ACFP, suppresses the growth of primary human ovarian cancer cells by regulating apoptotic gene expression and signaling pathways

**DOI:** 10.1186/s12885-016-2281-6

**Published:** 2016-03-24

**Authors:** Juan Zhou, Mengjing Zhao, Yigui Tang, Jing Wang, Cai Wei, Fang Gu, Ting Lei, Zhiwu Chen, Yide Qin

**Affiliations:** Department of Biochemistry and Molecular Biology, Anhui Medical University, Hefei, Anhui 230032 China; Department of Obstetrics and Gynecology, the First Affiliated Hospital of Anhui Medical University, Hefei, Anhui 230022 China; Department of Pharmacy, the Second Affiliated Hospital of Anhui Medical University, Hefei, Anhui 230601 China; Department of Pharmacology, Anhui Medical University, Hefei, Anhui 230032 China

**Keywords:** Fusion peptide, Anti-ovarian cancer, Cell viability, Apoptosis, cDNA microarray

## Abstract

**Background:**

ACFP is an anti-cancer fusion peptide derived from bovine milk protein. This study was to investigate the anti-cancer function and underlying mechanisms of ACFP in ovarian cancer.

**Methods:**

Fresh ovarian tumor tissues were collected from 53 patients who underwent initial debulking surgery, and primary cancer cells were cultured. Normal ovarian surface epithelium cells (NOSECs), isolated from 7 patients who underwent surgery for uterine fibromas, were used as normal control tissue. Anti-viabilities of ACFP were assessed by WST-1 (water-soluble tetrazolium 1), and apoptosis was measured using a flow cytometry-based assay. Gene expression profiles of ovarian cancer cells treated with ACFP were generated by cDNA microarray, and the expression of apoptotic-specific genes, such as *bcl*-xl, *bax*, *akt*, *caspase*-3, *CDC*25C and *cyclin*B1, was assessed by real time PCR and western blot analysis.

**Results:**

Treatment with ACFP inhibited the viability and promoted apoptosis of primary ovarian cancer cells but exhibited little or no cytotoxicity toward normal primary ovarian cells. Mechanistically, the anti-cancer effects of ACFP in ovarian cells were shown to occur partially via changes in gene expression and related signal pathways. Gene expression profiling highlighted that ACFP treatment in ovarian cancer cells repressed the expression of *bcl-*xl, *akt*, *CDC*25C and *cyclin*B1 and promoted the expression of *bax* and *caspase*-3 in a time- and dose-dependent manner.

**Conclusions:**

Our results suggest that ACFP may represent a potential therapeutic agent for ovarian cancer that functions by altering the expression and signaling of cancer-related pathways in ovarian cancer cells.

**Electronic supplementary material:**

The online version of this article (doi:10.1186/s12885-016-2281-6) contains supplementary material, which is available to authorized users.

## Background

Ovarian cancer remains the most lethal gynecologic tumor in the world. Serous cystadenoma is the most common histologic subtype of ovarian cancer. Although aggressive cytoreductive surgery followed by adjuvant chemotherapy (e.g., cisplatin and paclitaxel) has been shown to induce a clinical response in the majority of ovarian cancer patients, most women will eventually relapse and die due to the development of chemotherapy-resistant disease [1]. For this reason, there is a strong need novel treatment options in this patient population. Peptide therapeutics represents an emerging field of anti-cancer agents that are easily obtained from either natural resources or are designed based on target protein structure. Moreover, past reports indicate that therapeutic peptides typically exhibit little toxicity in normal host cells [2]. Recently, Su L. Y. et al. [3] reported that the anti-cancer bioactive peptide (ACBP), purified from goat spleens that were immunized with human gastric cancer extracts, significantly inhibited gastric cancer cell growth in vitro and gastric tumor growth in vivo, indicating that therapeutic peptides may represent a powerful anti-cancer tool.

Biologically active peptides have also been described in anti- ovarian cancer cells, including LfcinB (bovine lactoferricin), a peptide originally derived from bovine lactoferrin, which has been shown significantly to inhibit the in vitro growth and in vivo tumor development of the ovarian cancer cell line SKOV3 [4]. LfcinB is derived in bovine lactoferrin (sequence of 17–41 residues), having 25 amino acids (FKCRRWQWRMKKLGAPSITCVRRAF). According to the literature, the active site of LfcinB residues in residues 4 to 9 of the amino acid sequence [5, 6]. However, clinical use of LfcinB is most likely limited due to high toxicity, poor stability and the propensity of the molecule to undergo structural changes under different environments that affect the biological activity of LfcinB. Although the anti-cancer effects of LfcinB have been generally accepted, the underlying mechanisms of LfcinB function remain unclear.

The hexapeptide (Pro-Gly-Pro-Ile-Pro-Asn, PGPIPN, 63–68 residues of bovine ß-casein), also known as immune hexapeptide or immunomodulating peptide, was isolated from hydrolysate of bovine β casein and has been shown to elicit an immune response in cancer cells [7–10]. In line with this finding, our research group recently showed that PGPIPN inhibited the growth of SKOV3 cells in vitro and promoted apoptosis and decreased tumor growth in a xenograft ovarian cancer model [11]. However, the anti-cancer effects of PGPIPN are significantly lower than those of classical anti-cancer drugs, such as paclitaxel or 5-fluorouracil (5-FU) [11].

Based on structure-function studies of LfcinB and PGPIPN, we performed molecular modification of the two biological peptides derived from milk proteins. Using web- (http://swissmodel.expasy.org) and software-based (Accelery Insight II 2005.1LBIOVIA, San Diego, USA) tools, we designed an anti-cancer fusion peptide (ACFP) that connects regions of LfcinB and PGPIPN by a flexible link arm (GGGGS). Importantly, fusion of the two peptides led to a molecule with superior anti-cancer function and increased overall structural stability and anti-enzymatic hydrolysis. ACFP consists of a disulfide bond chain, which is likely to lead to cancer cell membrane localization. The theoretical molecular weight of ACFP was calculated to be 3960 (our experimental determination is 4020), which is smaller than the immunogenic molecules and does not stimulate immunogenicity.

In this study, we investigated the anti-viabilities of ACFP in primary ovarian cancer cell and normal ovarian epithelial cells in vitro. Using cDNA chip, we observed that ACFP treatment led to significant changes in gene expressions and cancer-associated signaling pathways in primary ovarian cancer cells. ACFP-specific effects on *bcl*-xl, *bax*, *akt*, *caspase*-3*, CDC*25C and *cyclin*B1 gene expressions were confirmed using real time PCR and western blot analysis. Overall, this study investigates the molecular mechanisms that underlie the anti-viabilities of ACFP on anti-ovarian cancer and provides the experimental basis for developing ACFP as a new therapeutic agent in ovarian cancer.

## Methods

### Reagents

The ACFP peptide was provided by Shanghai Sangon Biological Engineering Technology, and the purity was confirmed by RP-HPLC to be > 99.5 %. Trizol Kit was purchased from Invitrogen, USA. Reverse Transcription System was purchased from Promega, USA. Eight joint tubes for PCR were purchased from ABI, USA. SYBR Green I Premix Ex Taq was purchased from Takara Biotechnology (Dalian) Co., Ltd, China. Mouse monoclonal antibodies of Bcl-xl, Bax, Akt, Caspase-3, CDC25C, CyclinB1 and β-Actin were purchased from Santa Cruz Biotechnology, Inc. The horseradish peroxidase conjugated secondary antibody (goat anti-mouse IgG) and Super Signal West Pico Trial Kit (ECL chromogenic reagent kit) were purchased from Pierce, USA; BCA Kit for protein quantitative assay was purchased from Shanghai Sangon Biological Engineering Technology.

### Cell cultures

Fresh primary ovarian tumor tissues that were assessed and classified as serous ovarian adenocarcinoma (III-IV grade) according to WHO criteria were collected from 53 ovarian cancer patients who underwent initial debulking surgery at the first affiliated hospital of Anhui Medical University between June 2012 and June 2014. All patients had not received adjuvant therapies, such as chemotherapy or radiotherapy, prior to surgery. For comparison, normal ovarian glandular epithelium cells (NOGECs) were cultured from fresh primary normal ovarian tissues harvested from 7 patients with uterine fibromas, which were confirmed as negative for any neoplastic disease by pathological examination. Prior to tissue deposition, all patients signed written consent forms confirming their donation of tissue for research purposes according to the Declaration of Helsinki. This study was approved by the Anhui Medical University Review Board. Tumor or normal ovarian tissues were cut into small 1.0 mm^3^ pieces, rinsed two times in phosphate buffered saline (PBS) and digested with 0.25 % trypsin in a sterile centrifuge tube at 37 °C for 30 min. To obtain a single cell suspension cell, digested tissues were filtered with a 100 μm cell strainer. Cells were collected by centrifugation at 1000 rpm for five minutes, and the cell pellet was re-suspended in Dulbecco’s modified eagle medium (DMEM) supplemented with 10 % fetal bovine serum (FBS). Cells were subsequently cultured in DMEM containing 0.1 mg/L epidermal growth factor (EGF), 0.1 mg/L insulin-like growth factor (IGF) and 0.1 mg/L beta-estradiol with 10 % FBS in 5 % CO_2_ at 37 °C. Once cells reached 70 to 80 % confluence, cell culture medium was drained from the flask, and cells were digested with 0.25 % collagenase II until approximately 1/3 of the cells fell to the bottom of the dish by eye using a microscope. Due to their initial shedding, most fibroblasts were eliminated by collagenase digestion. The remaining cells were cultured in 5 % CO_2_ at 37 °C. To determine purity, cells were analyzed using immunofluorescence of cytokeratin 7 (for ovarian cancer cells) or cytokeratin 19 (for normal ovarian epithelium cells).

### Cell viability assay

Primary ovarian cancer cells were seeded into 96-well plates in sextuplicate at a starting density of 5 × 10^3^ cells/well and incubated with ACFP at the concentrations: 0 (as control), 5 × 10^−6^, 5 × 10^−5^, 5 × 10^−4^, 5 × 10^−3^, 5 × 10^−2^, 5 × 10^−1^ and 5 g/L for 24, 48 and 72 h, respectively. Cells treated with paclitaxel at 5 × 10^−4^ g/L were included in the same plate as a positive control. Cell viability was later measured using the WST-1 (water-soluble tetrazolium 1) cell viability and cytotoxicity assay kit (Beyotime, Haimen, China) according to the manufacturer’s instructions. The percent viability of cells was calculated using the formula to calculate the cell viability ratio (VR): VR (%) = (the experimental group A_450nm_ value/control group A_450nm_ value) × 100 %. To assess general toxicity of ACFP, viability of normal ovarian cells treated with ACFP was assayed using the same procedure. The effect of ACFP was compared with LfcinB and PGPIPN (the products of Shanghai Sangon Biological Engineering Technology, China). Each experiment was performed in two independent sets.

### Apoptosis assay

Apoptosis of primary ovarian cancer cell treated with ACFP was measured by flow cytometry (FCM) using FITC-conjugated Annexin-V and propidium iodide (PI) from Sigma. Cells were washed twice with cold PBS and resuspended in Annexin-V binding buffer (10 mM HEPES, 140 mM NaCl and 5 mM CaCl_2_) at a concentration of 1 × 10^6^ cells/mL. A single cell suspension of 1 × 10^6^ cells was prepared in a 5 mL culture tube according to the instrument manual and 5 μL Annexin-V-FITC at 10 μg/mL and 10 μL propidium iodide at 10 μg/mL was added. The tube was gently vortexed and incubated for 15 min at room temperature in the dark. Binding buffer (400 μL) was subsequently added to each tube and the cells were analyzed by flow cytometry (EPICSR XL-MCL, Beckman, USA) with EXPO32TM ADC software (Beckman, USA). Cells that stained positive for annexin V were counted as apoptotic.

### cDNA microarrays in screening of differentially expressed genes

We utilized cDNA microarrays to observe the effect of ACFP on gene expression in primary ovarian cancer cells. Cells were treated with 0 (the vehicle group, as control), 5 × 10^6^ g/L and 5 × 10^3^ g/L ACFP, respectively, and cultured for 48 h at 37 °C in 5 % CO_2_. Cells were later digested and collected for total RNA extraction using Trizol. The RNA concentrations and purities were detected with a spectrophotometer. The experiments were performed in duplicate on a single total RNA preparation from the cells. Signal values were presented as the mean value of two replicate experiments. RNA samples were used to generate human whole genome expression profiling microarray (Yeli Bioscience Co., Ltd.; Shanghai, China). RNA was subsequently converted into digoxigenin-labeled complementary RNA and hybridized to a human genome microarray system (Human OneArray Microarray, from Phalanx Biotech Group, Taiwan). The chips were scanned by a GeeDom® LuxScan 10 K microarray scanner. LuxScan3.0 software was used to extract probe fluorescence signals and analyze images. Finally, the original data were processed by normalizing. The differential gene screening, cluster analysis and pathway analysis were conducted from microarray data by Shanghai Sensichip Infotech Co. Ltd. (China). Genes that displayed a signal value greater than 100 and a ratio of ACFP treatment vs control greater than 2 were defined as up-regulated, while genes with a signal value greater than 100 and a ratio of ACFP treatment vs control less than 0.5 were defined as down-regulated.

### Real time PCR in measuring mRNA of *bcl-xl*, *bax*, *akt*, *caspase*-3, *CDC*25C and *cyclin*B1

An optimized RT-PCR protocol was employed to analyze the mRNA levels of *bcl*-xl, *bax*, *akt*, *caspase*-3, *CDC*25C and *cyclin*B1. Beta-*actin* was used as a housekeeping gene. According to primer sequences of *bcl*-xl, *bax*, *akt, caspase*-3, *CDC*25C, *cyclin*B1 and β-*actin* genes retrieved from Primer-Bank, primers were designed with the Primer 5.0 software, which were synthesized by Shanghai Sangon Biological Engineering Technology. These primer sequences are as follows:

- *bcl*-xl forward 5′-AGCTGGTGGTTGACTTTCTCTC-3′,
- *bcl*-xl reverse 5′-CCTCAGTCCTGTTCTCTTCCAC-3′;
- *bax* forward 5′-GGTTGTCGCCCTTTTCTACTTT-3′,
- *bax* reverse 5′-GTGAGGAGGCTTGAGGAGTCT-3′;
- *akt* forward 5′-CGGGGTAGGGAAGAAAACTATC-3′,
- *akt* reverse 5′-TGACAGAGTGAGGGGACACA-3′;
- *caspase-*3 forward 5′-GACTCTGGAATATCCCTGGACAACA-3′,
- *caspase*-3 reverse 5′AGGTTTGCTGCATCGACATCTG-3′;
- *CDC*25C forward 5′-GCTAACAAGTCACCAAAAGACA-3′,
- *CDC*25C reverse 5′-TCCCTGAACCAATACAATCTC-3′;
- *cyclin*B1 forward 5′-AGGTCCATCTCAGGTTCCACTT-3′,
- *cyclin*B1 reverse 5′-GAGTAGGCGTTGTCCGTGAT-3′;
- β-*actin* forward 5′-ATGTTTGAGACCTTCAACACCCC-3′,
- β-*actin* reverse 5′-GCCATCTCTTGCTCGAAGTCCAG-3′.

Primary ovarian cancer cells were harvested after ACFP treatment at different doses and times, respectively. The total RNAs in the primary ovarian cancer cells were extracted according to the Trizol kit manufacturer’s instructions, and the purity and concentration were determined by ultraviolet spectrophotometry. According to the RNA template and primers, cDNAs of specific genes were synthesized in system of reverse transcription reaction including 10 × 2 μl buffers, dNTPs (10 mM) 2 μl, AMV reverse transcriptase of 1 μl, 0.5 μl recombinant RNasin and total RNA 1 μl in final volume of 20 μl by adding RNase-free water. The reverse transcription reaction conditions were 42 °C 15 min and 95 °C 5 min. After the reaction, the reverse-transcribed cDNAs were diluted with RNase-free water to a final volume of 60 μL and preserved at 80 °C.

Real time PCR adopts TaKaRa SYBR Green as real time PCR Master Mix in ABI7500 fluorescent real-time PCR instrument. The reaction conditions were as follows: 95 °C × 30 s (1 cycle); 95 °C × 5 s, 60 °C × 34 s (40 cycles). At the end of PCR cycling steps, data for each sample were displayed as a melting curve. The specificity of the amplified products was confirmed using melting curve analysis. The ABI SDS software (Applied Biosystems) was used to determine a critical threshold (C_t_), which was defined as the cycle number where the linear phase for each sample crossed the threshold level. The mRNAs of target gene expression were denoted by ΔC_t_ (ΔC_t_ = target gene C_t_ - β-*actin* C_t_ value). Finally, the relative mRNA expression of all samples were calculated using the 2^-ΔΔC^_t_ method [12]. All reactions were performed in triplicate, and a mixture lacking a complementary DNA template (NTC) was used as the negative control.

### Western blot for analysis of Bcl-xl, Bax, Akt, Caspase-3, CDC25C and CyclinB1 proteins

Proteins were isolated from primary ovarian cancer cells harvested after ACFP treatment, separated by SDS-PAGE and transferred to PVDF membrane using the standard protocol. After blocking with 5 % (w/v) dry skim milk, membranes were incubated with primary antibodies (mouse monoclonal Bcl-xl, Bax, Akt, Caspase-3, CDC25C, CyclinB1 and β-Actin antibodies, 1:1000 dilution) according to the manufacturer’s instructions and later incubated with a horseradish peroxidase conjugated secondary antibody (goat anti-mouse IgG, 1:8000 dilution). The proteins were detected with the enhanced chemiluminescence (ECL) system followed by exposure to X-ray film. The β-Actin was used as a loading control. Two independent experiments were performed. Digital images were captured by Gel DocTM gel documentation system (Bio-Rad, USA) and intensities were quantified using Quantity-One software version 4.62 (Bio-Rad, USA).

### Statistical analysis

All data were expressed as the mean ± SD. The differences among groups were analyzed using the one-way ANOVA by SPSS 15.0 statistical software. The results were considered to be statistically significant when *P* < 0.05.

## Results

### ACFP structure was predicted by bioinformatics

**Fig. 1:**
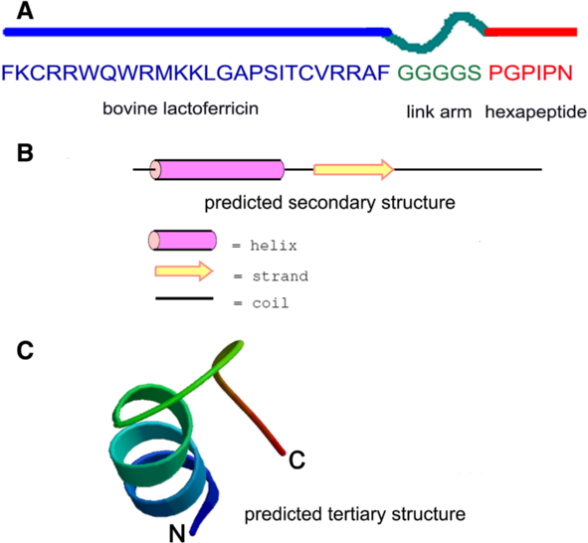
Design and structure analysis of the anti-cancer fusion peptide (ACFP). **a** The design and framework of ACFP generated from LfcinB and PGPIPN sequences. **b** The predicted secondary structure of ACFP (http://swissmodel.expasy.org). **c** The predicted tertiary structure of ACFP (http://bioinf.cs.ucl.uk/pripred)

According to our design, the primary structure of ACFP is shown in Fig. 1a.

Using bioinformatics analysis (http://bioinf.cs.ucl.uk/pripred), the predicted secondary and tertiary structures of ACFP are shown in Fig. 1b and c. According to bioinformatics analysis, the activity centers of LfcinB and PGPIPN are not damaged following fusion.

### ACFP inhibited the viability of human primary ovarian cancer cells

**Fig. 2:**
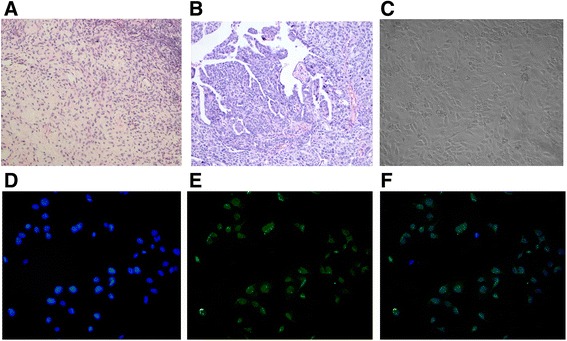
Culturing of primary human ovarian cancer cells. **a** Pathological section of normal human ovarian tissue with benign pathology (H&E stained, ×100). **b** Pathological section of human ovarian cancer tissue (H&E stained, ×100) that was classified as serous ovarian adenocarcinoma (I-II grade) according to WHO criteria. **c** Representative morphology of ovarian carcinoma cells grown in primary culture medium (×100). **d** Cultured primary human ovarian cancer cells stained with nuclear dyes-Hochest33258 (×100). **e** Cultured primary human ovarian cancer cells stained with anti-cytokeratin 7-FITC. **f** The confocal of D and E pictures

**Fig. 3:**
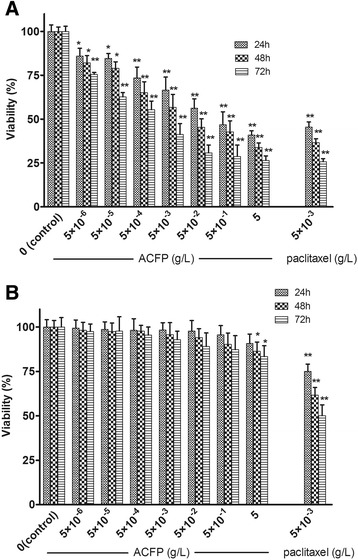
ACFP suppresses primary human ovarian cancer cells viability, but has little effect on untransformed cells. **a** Cell viability assay shows that ACFP treatment at different concentrations suppressed primary ovarian cell viability. Results are expressed as the mean ± SD of 53 primary ovarian cancer cell measurements from 53 patients, ^*^ *P* < 0.05, ^**^ *P* < 0.01 compared with control (the vehicle group). **b** ACFP had little or no effect on normal ovarian glandular epithelium cells (NOGECs) viability in vitro. Results are expressed as the mean ± SD of 7 primary normal ovarian cells for benign pathologies from 7 patients with uterine fibromas at initial debulking surgery, ^*^ *P* < 0.05, ^**^ *P* < 0.01 compared with control (the vehicle group)

We successfully isolated and established primary ovarian cancer cell lines from 53 ovarian cancer patients who underwent initial debulking surgery in the first affiliated hospital of Anhui Medical University. These primary cells were cultured in our laboratory and morphologically represent typical cancer cells. Immunocytochemistry analysis of anti-cytokeratin 7 staining (Fig. 2) revealed an average of approximately 84.61 % ovarian cancer cell purity within the isolated cell populations. To investigate whether ACFP affects primary ovarian cancer cell viability, cells were seeded 96-well plates, grown overnight and treated with differing concentrations of ACFP for 24, 48 and 72 h. As shown in Fig. 3a, treatment of ovarian cancer cells with ACFP led to a significant time- and dose-dependent decrease in cell viability. The anti-ovarian cancer activity of ACFP was significantly higher than its parent peptides (Additional file 1: Figure S1). The half maximal inhibitory concentrations (IC50s) of ACFP were 1.15 × 10^−2^, 1.63 × 10^−3^ and 3.88 × 10^−4^ g/L after 24, 48, and 72 h treatment, respectively, which were significantly lower than the IC50s calculated for LfcinB and PGPIPN. The IC50s of LfcinB were 4.11 × 10^−2^, 3.58 × 10^−3^ and 1.02 × 10^−4^ g/L after 24, 48, and 72 h treatment, respectively; and the IC50s of PGPIPN were 1.45 × 10^−2^, 9.30 × 10^−3^ and 1.24 × 10^−3^ g/L after 24, 48, and 72 h treatment, respectively. These results indicate that the primary ovarian cancer cells were sensitive to ACFP treatment. General cytotoxicity of ACFP on normal primary ovarian cells was also investigated using a WST-1 assay. Importantly, ACFP treatment exhibited little or no cytotoxicity toward untransformed cells compared with the traditional anti-cancer drug-paclitaxel (Fig. 3b).

### ACFP promoted apoptosis in human primary ovarian cancer cells

**Fig. 4:**
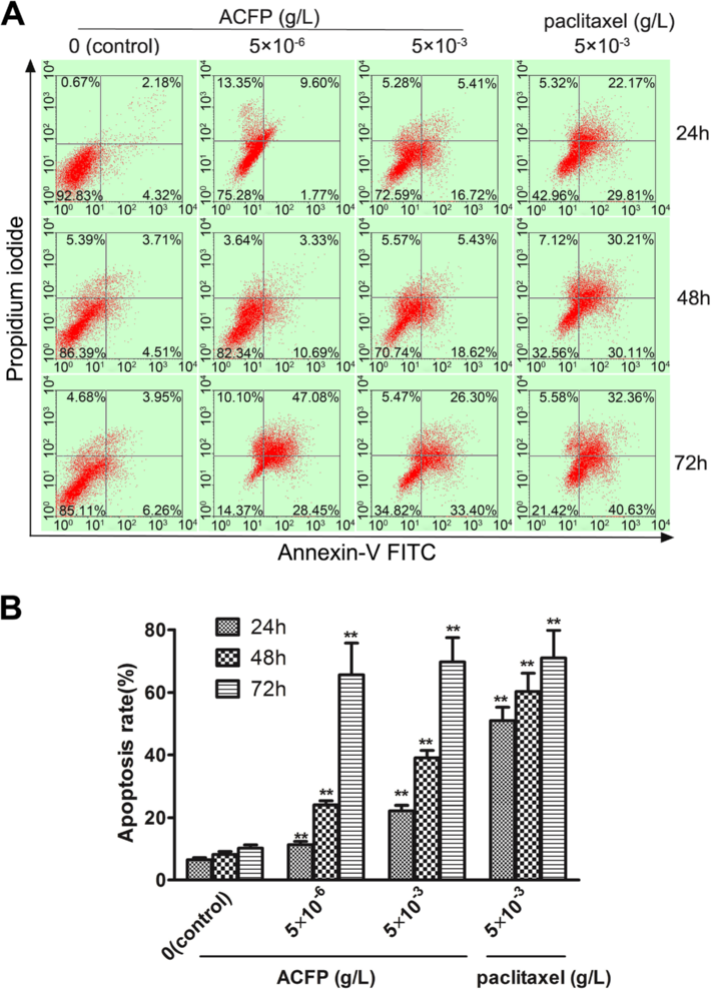
ACFP induces apoptosis in primary human ovarian cancer cells. **a** Representative flow cytometry dot plot of primary human ovarian cancer cells treated with ACFP and stained with Annexin-V-FITC and PI. **b** Histogram of apoptosis rates of primary human ovarian cancer cells treated with ACFP. The data are shown as means ± SD of 53 primary ovarian cancer cells measurements from 53 patients, ^*^ *P* < 0.05, ^**^ *P* < 0.01 compared with control (the vehicle group)

Using an Annexin V-TITC and PI double-staining method, ACFP treatment was shown to promote human primary ovarian cancer cell apoptosis *in vitro* (Fig. 4) in a time- and dose- dependent manner.

### cDNA microarrays revealed differentially expressed genes in human primary ovarian cancer cell treated with ACFP

**Fig. 5:**
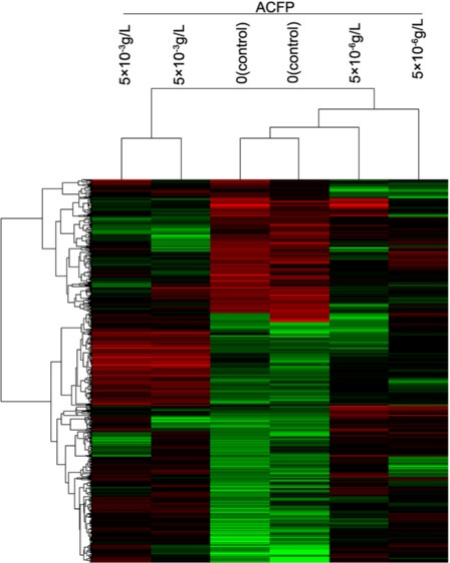
Hierarchical cluster analysis of differentially expressed genes in primary human ovarian cancer cells treated with ACFP. Hierarchical cluster analysis included 6 samples (2 controls, 2 ACFP-treateds at 5 × 10^−6^g/L and 2 ACFP-treateds at 5 × 10^−3^g/L) that were treated for 48 h. Each column represents a gene, each row represents a sample; red: high expression level, green: low expression level, black: unchanged expression

Compared with the control condition, 744 genes were found differentially expressed in cells treated with a low dose of ACFP (5 × 10^6^ g/L ACFP), including 486 up-regulated and 258 down-regulated genes, as shown Fig. 5. Similarly, 1177 genes were found differentially expressed in cells treated with a high dose of ACFP (5 × 10^3^ g/L ACFP), of which 791 genes were up-regulated and 386 genes were down-regulated (Fig. 5). Among them, genes related to apoptosis were listed in Tables 1 and 2, all *P*-values in their gene array tables were less than 0.05 or 0.01. Pathway analysis of the most differentially regulated genes highlighted such cell processes as apoptosis, cell cycle, chemokine and other signaling pathways (Table 3).

**Table 1:** The up-regulated expression profiling of genes related to apoptosis in ACFP-treated human primary ovarian cancer cells in vitro

Genebank ID	Gene symbol	Gene description	Ratio (ACFP/control)	
			Low dose	High dose
NM_000581	BAX	BCL2-associated X protein	3.73	6.35
NM_000836	CASP3	caspase 3, apoptosis-related cysteine peptidase	3.72	5.34
NM_008106	PABPN1	poly(A) binding protein, nuclear 1	3.71	9.8
NM_001164	CKS2	CDC28 protein kinase regulatory subunit 2	3.67	8.95
NM_007920	BAT5	HLA-B associated transcript 5	3.66	7.48
NM_010653	SPINT2	serine protease inhibitor, Kunitz type, 2	3.58	10.25
NM_001855	DVL1	dishevelled segment polarity protein 1	3.58	2.46
NM_114904	C1QTNF6	C1q and tumor necrosis factor related protein 6	3.57	6.26
NM_002949	GSTM5	glutathione S-transferase M5	3.55	4.84
NM_007132	TNFRSF1A	tumor necrosis factor receptor superfamily, member 1A	3.50	2.15
NM_007132	TNFRSF1A	tumor necrosis factor receptor superfamily, member 1A	3.50	2.08
NM_055970	GNG12	guanine nucleotide binding protein (G protein), gamma 12	3.49	8.30
NM_009736	USP34	ubiquitin specific protease 34	3.49	4.52
NM_441241	LOC441241	similar to chaperonin containing TCP1, subunit 6A (zeta 1); chaperonin containing T-complex subunit 6	3.48	5.22
NM_114904	C1QTNF6	C1q and tumor necrosis factor related protein 6	3.43	6.26
NM_051275	FLJ39616	apoptosis-related protein PNAS-1	3.34	2.53
NM_009618	TRAF4	TNF receptor-associated factor 4	3.34	3.62
NM-016522	TMEFF1	transmembrane protein with EGF-like and two follistatin-like domains 1	3.33	7.91
NM_079829	FLJ13848	hypothetical protein FLJ13848	3.28	9.45
NM-009774	BTF/BCLAF1	BCL2-associated transcription factor 1	3.25	2.79
NM-006777	STAT5B	signal transducer and activator of transcription 5B	3.24	2.03
NM_001026	CDKN1A	cyclin-dependent kinase inhibitor 1A (p21, Cip1)	3.24	6.58
NM_001026	CDKN1A/p21	cyclin-dependent kinase inhibitor 1A (p21, Cip1)	3.24	3.58
NM_003665	IRF7	interferon regulatory factor 7	3.13	4.50
NM_081788	SNARK	likely ortholog of rat SNF1/AMP-activated protein kinase	3.08	3.11
NM_000390	ARHE	ras homolog gene family, member E	2.99	3.39
NM_005696	PSMB8	proteasome (prosome, macropain) subunit, beta type, 8 (large multifunctional protease 7)	2.97	3.24
NM_005603	MAPK13/p38 delta	mitogen-activated protein kinase 13	2.91	3.04
NM_010209	SUI1	putative translation initiation factor	2.89	9.85
NM-004824	NKX3-1	NK3 transcription factor related, locus 1 (Drosophila)	2.8	3.11
NM_005569	PKIA	protein kinase (cAMP-dependent, catalytic) inhibitor alpha	2.52	2.74
NM_009950	GOLGA5	golgi autoantigen, golgin subfamily a, 5	2.46	2.95
NM_079370	BCL2L14	BCL2-like 14 (apoptosis facilitator)	2.44	2.57
NM_008795	TNFRSF10B	tumor necrosis factor receptor superfamily, member 10b	2.43	4.85
NM_029775	CARD10	caspase recruitment domain family, member 10	2.43	4.32
NM_002948	GSTM4	glutathione S-transferase M4	2.25	2.72
NM_079092	CARD14	caspase recruitment domain family, member 14	2.13	3.06
NM_007559	ZNF12	zinc finger protein 12 (KOX 3)	2.07	3.66
NM-006778	STAT6	signal transducer and activator of transcription 6, interleukin-4 induced	2.06	10.8
NM_000943	TNFRSF8	tumor necrosis factor receptor superfamily, member 8	2.05	3.22
NM_005599	MAPK8/JNK1	mitogen-activated protein kinase 8	2.04	2.74
NM_000943	TNFRSF8	tumor necrosis factor receptor superfamily, member 8	2.02	3.22
NM_000714	C1QG	complement component 1, q subcomponent, gamma polypeptide	2.02	2.68
NM_009262	STK17B	serine/threonine kinase 17b (apoptosis-inducing)	2.01	2.97

**Table 2:** The down-regulated expression profiling of genes related to apoptosis in ACFP-treated human primary ovarian cancer cell in vitro

Genebank ID	Gene symbol	Gene description	Ratio (ACFP/control)	
			Low dose	High dose
NM_000598	BCL-xl	bcl2-like 1	0.28	0.19
NM_000207	AKT1	v-akt murine thymoma viral oncogene homolog 1	0.29	0.27
NM_000995	CDC25C	cell division cycle 25C	0.29	0.32
NM_000891	CCNB1	cyclinB1	0.30	0.21
NM-022931	RAB18	RAB18, member RAS oncogene family	0.30	0.25
NM-084450	ZNF512	zinc finger protein 512	0.31	0.47
NM-060561	RINT-1	Rad50-interacting protein 1	0.32	0.17
NM-005429	POLH	polymerase (DNA directed), eta	0.33	0.12
NM-004638	MYLK	myosin, light polypeptide kinase	0.33	0.23
NM_063035	BCORL1	BCL6 co-repressor-like 1	0.35	0.25
NM-009448	MAP4K4	mitogen-activated protein kinase kinase kinase kinase 4	0.36	0.41
NM-051176	TCF/LEF1	lymphoid enhancer-binding factor 1	0.36	0.35
NM_005595	MAPK3/ERK1	mitogen-activated protein kinase 3	0.36	0.35
NM_003678	ITGA5	integrin, alpha 5 (fibronectin receptor, alpha polypeptide)	0.36	0.43
NM-005322	PLA2G5	phospholipase A2, group V	0.36	0.46
NM-084299	C17orf37	chromosome 17 open reading frame 37	0.39	0.38
NM-005682	PSMA1	proteasome (prosome, macropain) subunit, alpha type, 1	0.39	0.49
NM_023533	PIK3R5	phosphoinositide-3-kinase, regulatory subunit 5, p101	0.39	0.44
NM_002868	GRK4	G protein-coupled receptor kinase 4	0.42	0.37
NM-001544	CYP1A2	cytochrome P450, family 1, subfamily A, polypeptide 2	0.43	0.48
NM-003488	IGFBP5	insulin-like growth factor binding protein 5	0.45	0.23
NM-054984	PINX1	PIN2-interacting protein 1	0.45	0.47
NM-002970	GTF2IP1	general transcription factor IIi pseudogene 1	0.47	0.24
NM_002869	GRK5	G protein-coupled receptor kinase 5	0.47	0.47
NM_000573	BAG1	BCL2-associated athanogene	0.48	0.33
NM-007378	UPP1	uridine phosphorylase 1	0.48	0.42
NM_002870	GRK6	G protein-coupled receptor kinase 6	0.49	0.26
NM-008995	TNFSF18	tumor necrosis factor (ligand) superfamily, member 18	0.49	0.41

**Table 3:** The results of pathway analysis

Pathway name	*P* value (ACFP vs control)/gene number in pathway	
	Lower dose	High dose
Apoptosis	0.000971/12	0.000781/13
Chemokine signaling pathway	0.003907/20	0.001615/19
ErbB signaling pathway	0.030382/10	0.014949/11
mTOR signaling pathway	0.022528/7	0.020496/9
Insulin signaling pathway	0.037667/13	0.028081/14
Prostate cancer	0.038960/10	0.023229/12
Glutathione metabolism	0.039118/5	0.016997/6
beta-Alanine metabolism	0.040715/4	0.025722/5
Acute myeloid leukemia	0.041365/9	0.030514/11
VEGF signaling pathway	0.042746/11	0.0451753/9
Chronic myeloid leukemia	0.043575/10	0.043688/10
Cell cycle	0.044920/7	0.040853/7
Valine, leucine and isoleucine degradation	0.046396/7	0.041547/8
Glycerolipid metabolism	0.046614/7	0.042819/7
Fatty acid metabolism	0.047829/6	0.039429/7
Amyotrophic lateral sclerosis (ALS)	0.048708/7	0.045332/5
T cell receptor signaling pathway	0.049384/11	0.048102/11
B cell receptor signaling pathway	0.049921/8	0.040102/9
Regulation of actin cytoskeleton	0.049938/19	0.039208/14
Endometrial cancer	0.069635/8	0.049471/9
Pathways in cancer	0.101147/20	0.047471/25
Adipocytokine signaling pathway	0.104896/5	0.049791/7
MAPK signaling pathway	0.106127/17	0.047937/17
Epithelial cell signaling in Helicobacter pylori	0.115779/5	0.049997/6

### Real-time PCR confirmed ACFP-induced changes in apoptotic gene expression in primary ovarian cancer cells

**Fig. 6:**
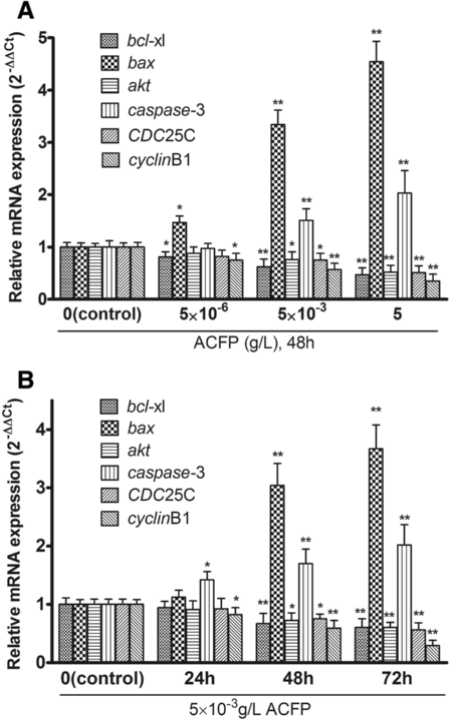
Real-time PCR validates ACFP-induced changes in *bcl*-xl, *bax*, *akt*, *caspase*-3, *CDC*25C and *cyclin*B1 mRNA levels in primary human ovarian cancer cells. **a** After ACFP treatment at different concentrations for 48 h, RNA was harvested from primary human ovarian cancer cells; real-time PCR using primers specific to *bcl*-xl, *bax*, *akt*, *caspase*-3, *CDC*25C and *cyclin*B1 was performed. **b** After 5 × 10^−3^ g/L ACFP treatment for different time, mRNA expressions of *bcl*-xl, *bax*, *akt*, *caspase*-3, *CDC*25C and *cyclin*B1 were detected in human primary ovarian cancer cells. The data in **a** and **b** are shown as means ± SD of 12 primary ovarian cancer cells measurements from 12 patients, ^*^ *P* < 0.05, ^**^ *P* < 0.01 compared with control (the vehicle group), taken β-*actin* as reference gene

Real-time PCR experiments were performed using *bcl*-xl-, *bax-*, *akt-, caspase*-3-, *CDC*25C- and *cyclin*B1-specific primers to assess their relative mRNA expressions (2^-ΔΔC^_t_) in human primary ovarian cancer cells treated with ACFP for 48 h (Fig. 6a). Increasing the concentration of drug was shown to promote a gradual increase in expression of *bax* and *caspase*-3, while the expression of *bcl*-xl, *akt*, *CDC*25C and *cyclin*B1 gradually decreased with increasing the concentration of drug (Fig. 6a). Similarly, with 5 × 10^−3^ g/L ACFP treatment 24, 48 and 72 h, the relative mRNA expressions (2^-ΔΔC^_t_) of *bax*, *bcl*-xl, *akt*, *caspase*-3, *CDC*25C and *cyclin*B1 are shown in Fig. 6b, the effect of which showed time dependent manner. Notably, ACFP-mediated effects on mRNA levels were more significant at later time points.

### ACFP promoted changes in protein levels of several differentially expressed genes related to apoptosis

**Fig. 7:**
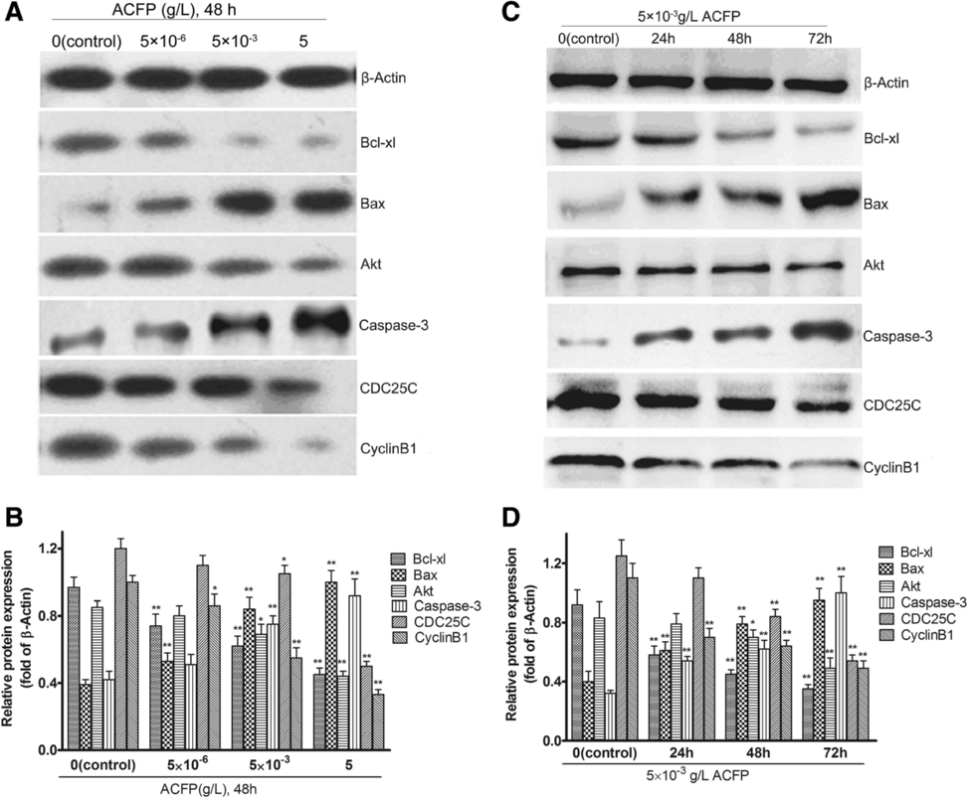
ACFP promotes changes in Bcl-xl, Bax, Akt, Caspase-3, CDC25C and CyclinB1 protein levels in primary human ovarian cancer cells. **a** Western blot analysis was performed after 48 h ACFP treatment in primary human ovarian cancer cells and antibodies specific to Bcl-xl, Bax, Akt, Caspase-3, CDC25C and CyclinB1 were used to assess protein levels. The β-Actin was used to show the similar amount of protein loaded in different lanes. **b** The relative intensities of protein bands in A were determined using Quantity-One software version 4.62 (Bio-Rad, USA) and normalized using β-Actin band intensity. **c** After 5 × 10^−3^ g/L ACFP treatment for different time, Bcl-xl, Bax, Akt, Caspase-3, CDC25C and CyclinB1 were detected. **d** The relative intensities of protein bands in **c** were determined using Quantity-One software and normalized using β-Actin band intensity. The data in **b** and **d** are shown as means ± SD of 12 primary ovarian cancer cells measurements from 12 patients, ^*^ *P* < 0.05, ^**^ *P* < 0.01 compared with control (the vehicle group)

Western blot analysis was used to demonstrate that treatment of human primary ovarian cancer cells with ACFP at different concentrations for 48 h led to dose-dependent changes in Bcl-xl, Bax, Akt, Caspase-3, CDC25C and CyclinB1 protein levels (Fig. 7a and b). Levels of Bax and Caspase-3 were determined to be elevated in ACFP-treated groups compared to control-treated group, while protein levels of Bcl-xl, Akt, CDC25C and CyclinB1 gradually decreased with increasing drug concentration. Notably, ACFP-mediated effects on protein levels were more significant at later time points (Fig. 7c and d).

## Discussion

Over the past 30 years, the slow improvement in the overall survival in high-grade serous ovarian cancer patients can be partly attributed to a lack of advancement in treatments beyond platinum-based combination chemotherapy [13]. Clinical application of anti-tumor chemical drugs is often limited due to frequent toxicity, narrow spectrum of activity and acquired resistance [14]. Thus, there is a need to explore or design new anti-tumor drugs with mechanisms of action that work, despite these obstacles. Among the newly developed anti-cancer drugs, bioactive peptides are one of the most promising drugs for the future of ovarian cancer therapy. Peptides are a novel class of anti-cancer agents that can be engineered to target cancer cells specifically with lower toxicity to normal tissues and offer new opportunities for cancer prevention and treatment [15]. Bioactive peptides are specific protein fragments that may have a positive impact on health and represent an important source of new anti-carcinogenic and immunomodulatory agents. Exploration of bioactive peptides plays a significant role in the development of innovative and unconventional anti-cancer drugs [16].

Milk is considered a nutritious food that consists of precursors of active peptides with biological and physiological properties. Bioactive peptides from milk proteins have been defined as specific protein fragments that have a positive impact on body functions or conditions and may ultimately influence health. The size of active milk peptides varies from 3 to 40 amino acid residues and many have been characterized as multi-functional proteins [17]. A human milk peptidomics study conducted by Dallas D.C. et al. [18] identified over 300 peptides by mass spectrometry analysis, of which the majority consisted primarily of peptides derived from β-casein and a large number of peptides that showed significant sequence overlap with peptides with known functions. Bovine milk proteins are currently the primary source of a range of biologically active peptides derived in milk, and among milk-born bioactive peptides, anti-cancer peptides have been exhibited a broad potential for clinical application in human trials and clinical studies. For example, treatment of biliary cancer patients with probiotic ingestion combined with radiotherapy led to significantly greater tumor regression and increased overall survival compared to radiotherapy treatment alone [19].

Peptides possess many advantages for the development of anti-tumor medications, including high selectivity, high potency, a broad range of targets, and low toxicity. However, poor stability, low membrane permeability, and susceptibility to proteolytic digestion have limited their clinical use to date [15]. To overcome these obstacles, modifications of peptide structure should be feasible and may lead to increased bioactivity. For example, development of flexible fusion peptides may lead to greater access into the cell and therefore more efficient disruption of targeted pathways [20]. Currently, a number of modification strategies have been developed and have been successfully used to improve the efficiency of anti-cancer peptides.

In the present study, we developed an anti-cancer fusion peptide based on sequences from LfcinB from bovine lactoferrin and hexapeptide (PGPIPN) from bovine β casein. LfcinB was chosen because its anti-tumor activity has recently been established in several cell lines and *in vivo* models, as demonstrated by membrane disruption and extensive hemorrhagic necrosis, respectively. Importantly, LfcinB peptides that harbor cationic residues within one sector of the helical structure were shown to be the most active in tumor cell lines, suggesting that specific structural regions are linked with bioactivity [21]. Despite its anti-tumor activity, issues with LfcinB stability and susceptibility to proteases have prevented its clinical use. In contrast to LfcinB, the PGPIPN peptide is rich in proline residues, rendering the molecule resistant to proteolytic degradation [21]. However, PGPIPN activity is lower than that of the traditional anti-cancer drugs, such as paclitaxel and cisplatin. Compared with its parent peptides, ACFP had many advantages. According to bioinformatics, the peptide has a disulfide bond and a stable α-helix in N-terminal, which is a relatively stable molecular. The peptide C-terminal containing three prolines can resist the hydrolysis of proteaseto [21]. Our previous experiments also showed that the peptide was very stable (Additional file 2: Figure S2). ACFP contains 8 charged amino acids (three lysines, five arginines) and can easily dissolve in water. ACFP is slightly soluble in fat (octanol). According to our preliminary experiments, oil (octanol)/water partition coefficient of the peptide was −0.91 (pH 7). From the experimental results, the ACFP peptide is superior to LfcinB and PGPIPN with increased structural stability, anti-cancer activity, intracellular access and reduced toxicity in normal cells.

Genomic characterization of ovarian cancers has emphasized the role of gene mutation and/or altered gene expression in the initiation and progression of the disease. In this study, we show using cDNA microarray that ACFP can up-regulate the expression of certain genes (including *bax* and *caspase*-3), and down-regulate the expression of certain other genes (including *bcl*-xl, *akt*, *CDC*25C and *cyclin*B1). Building on these results, we hypothesize that screening a larger panel of gene expression profiles from human ovarian cancer cells will most likely help to elucidate how ACFP induces anti-tumor activity [22]. Among the most differentially regulated genes, many are related to cell viability, cycle and apoptosis. However, we recognize that cDNA microarray results may include false positives. Therefore, we validated these results further using real-time PCR and western blot analysis and confirmed that apoptotic-related genes were regulated at the mRNA and protein levels in response to ACFP treatment [23].

The Bcl protein family plays an important role in regulating the apoptotic cell response. The Bcl proteins family is comprised of two classes: anti-apoptotic proteins (e.g., Bcl-xl) and pro-apoptotic proteins (e.g., Bax). Bcl-xl has been proposed as an oncogene based on its ability to block cell apoptosis and trigger tumorigenesis, while other proteins, such as Bak, are inhibitors of cell apoptosis [24]. Importantly, other survival factors, such as Akt, can also suppress apoptosis in a transcription-independent manner by phosphorylating and inactivating components of the apoptotic machinery. Akt can prevent cell apoptosis by inhibiting and modifying Bcl-xl. Akt is associated with tumor growth through PI3K/Akt pathway by targeting IGF and/or IGF receptor [25, 26]. Caspase-3 represents a core member of the apoptosis cascade pathway and is referred to as a death protease [27]. Caspase-3 is the most important effector of cell apoptosis and is located downstream of executing proteases in the caspase cascade. The *CDC*25C and *cyclin*B1 genes are also related to cell apoptosis. One of the hallmarks of cancer is the lack of regulation in the cell cycle. The *CDC*25C gene encodes for a tyrosine phosphatase protein that belongs to the Cdc25 phosphatase family and plays a key role in the regulation of cell division. CDC25C directs dephosphorylation of cyclin B-bound CDC2 (CDK1) and triggers entry into mitosis [28]. Overexpression of cyclinB1 can lead to uncontrolled cell growth by deregulation binding and activation of cell cycle activating CDK kinases. Binding of Cdks can lead to phosphorylation of other substrates at inappropriate time and unregulated viability [29].

In this study, ACFP can repress mRNA and protein expression of *bcl*-xl, *cyclin*B1, *CDC*25C and *akt* and enhance mRNA and protein expression of *bax* and *caspase*-3 by real time PCR and western blot analysis. The changes of *bcl*-xl, *bax, akt*, *cyclin*B1, *CDC*25C and *caspase*-3 expressions by real time PCR and western blot analysis were consistent with cDNA microarray results. Differential regulation of these pathways suggests that ACFP inhibits ovarian cancer cell growth by inhibiting anti-apoptotic protein Bcl-xl, Akt, CDC25C and cyclinB1 and promoting pro-apoptotic protein Bax and Caspase-3.

In the present study, we assessed the overall ACFP anti-cancer activity in primary ovarian cancer cells from ovarian tumor tissue of patients and analyzed ACFP effects on cancer-related gene expression and signal transduction pathways to determine whether transcription-related changes were related to ACFP-mediated anti-viabilities in primary ovarian cancer. However, we have not fully understood how the milk-derived fusion peptide bound the target cells. Fiedorowicz E, et al. reported that three bioactive peptides (opioid peptides) isolated from bovine caseins influenced the viability and cytokine secretion of human peripheral blood mononuclear cells by binding the μ-opioid receptor on the cytomembrane [30]. In our previous studies, we observed that hexapeptide (PGPIPN) isolated from bovine β-casein reduced Bcl-2 expression and induced cell apoptosis by binding target molecules on SKOV3 [11]. In the future, we plan to investigate whether ACFP binds cell specific receptor(s) in ovarian cell and, if so, determine the specific ACFP target molecule. We acknowledge that in our study, the number of ovarian tumor tissue samples (53 patients) and normal ovarian tissue samples (7 patients) was limited, emphasizing the need for future studies to extend on these findings. Together, our results provide a rationale for the future development of potent therapeutic peptides for the treatment of ovarian cancer.

## Conclusion

An anticancer fusion peptide (ACFP) inhibited the cells viabilities and induced the cells apoptosis of human primary ovarian cancer in vitro. The cDNA microarray showed ACFP affected genes expressions and signal pathways. The *bcl-*xl, *bax akt*, *caspase*-3, *CDC*25C and *cyclin*B1 genes expressions were identified by real time PCR and western blot. In conclusion, ACFP is a potential therapeutic agent for human ovarian cancer.

## Additional files

Additional file 1: Figure S1. ACFP, PGPIPN, LfcinB and mixture (PGPIPN+LfcinB) decreased growth of human primary ovarian cells at different concentrations for 48 h *in vitro*. The data are shown as means ± SD (*n*=12), **P*<0.05, ***P*<0.01 compared with control. (TIF 203 kb) Additional file 2: Figure S2. ACFP had good stability in pH 7 water. The data are shown as means ± SD (*n*=3). (TIF 117 kb)

## Abbreviations

ACFP: anti-cancer fusion peptide
DMEM: Dulbecco’s modified eagle medium
FBS: fetal bovine serum
FCM: flow cytometry
LfcinB: bovine lactoferricin
NOGECs: normal ovarian glandular epithelium cells
PBS: phosphate buffered saline
WST-1: water-soluble tetrazolium 1
