# Supplementary figures and images for: The milk-derived fusion peptide, ACFP, suppresses the growth of primary human ovarian cancer cells by regulating apoptotic gene expression and signaling pathways
Source: BMC Cancer. 2016 Mar 24;16:246. doi: 10.1186/s12885-016-2281-6 (PMC4806491; doi:10.1186/s12885-016-2281-6)

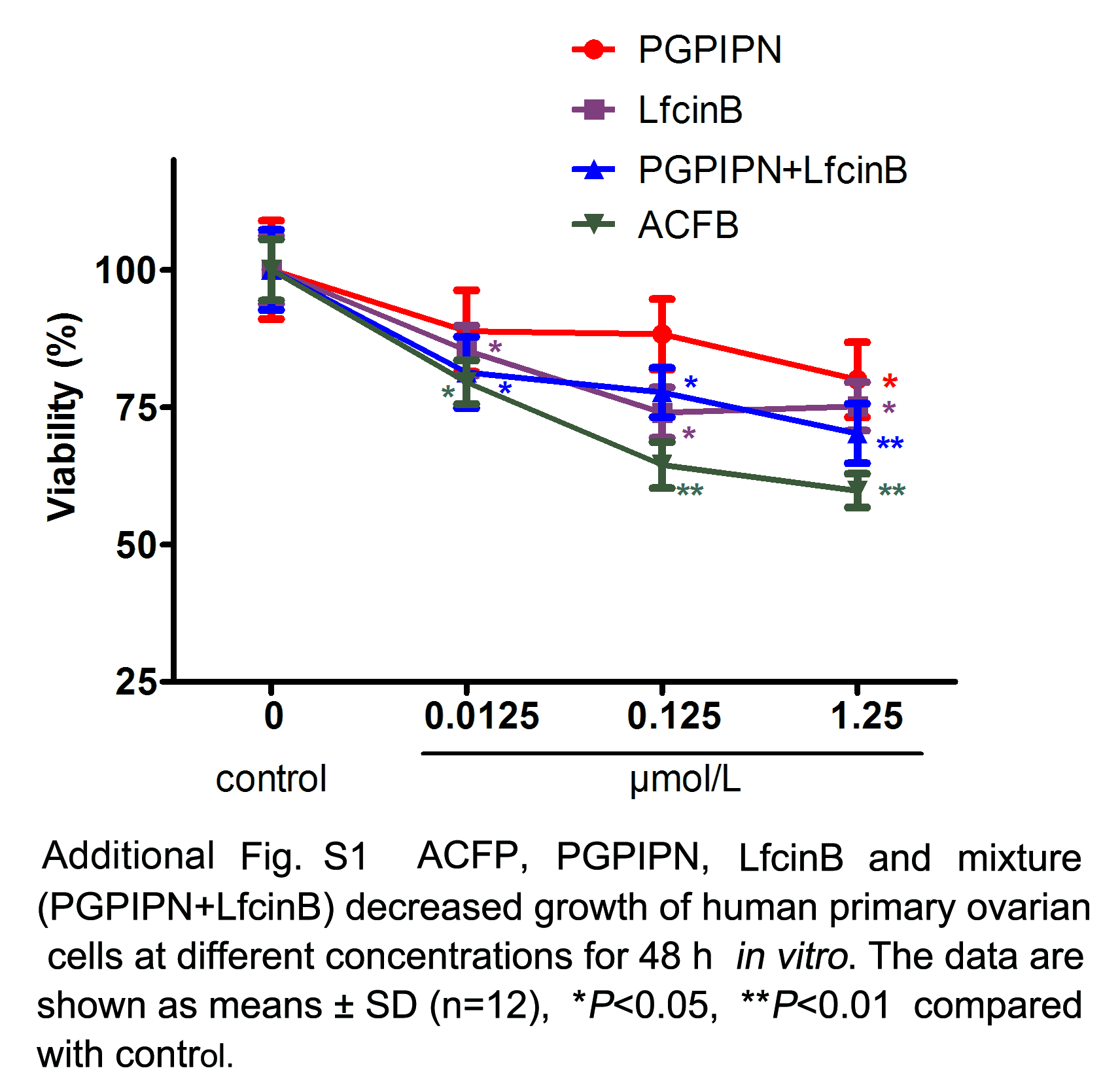

Supplement: Additional file 1: Figure S1. — ACFP, PGPIPN, LfcinB and mixture (PGPIPN+LfcinB) decreased growth of human primary ovarian cells at different concentrations for 48 h in vitro. The data are shown as means ± SD (n=12), *P<0.05, **P<0.01 compared with control. (TIF 203 kb) [file 12885_2016_2281_MOESM1_ESM.tif]

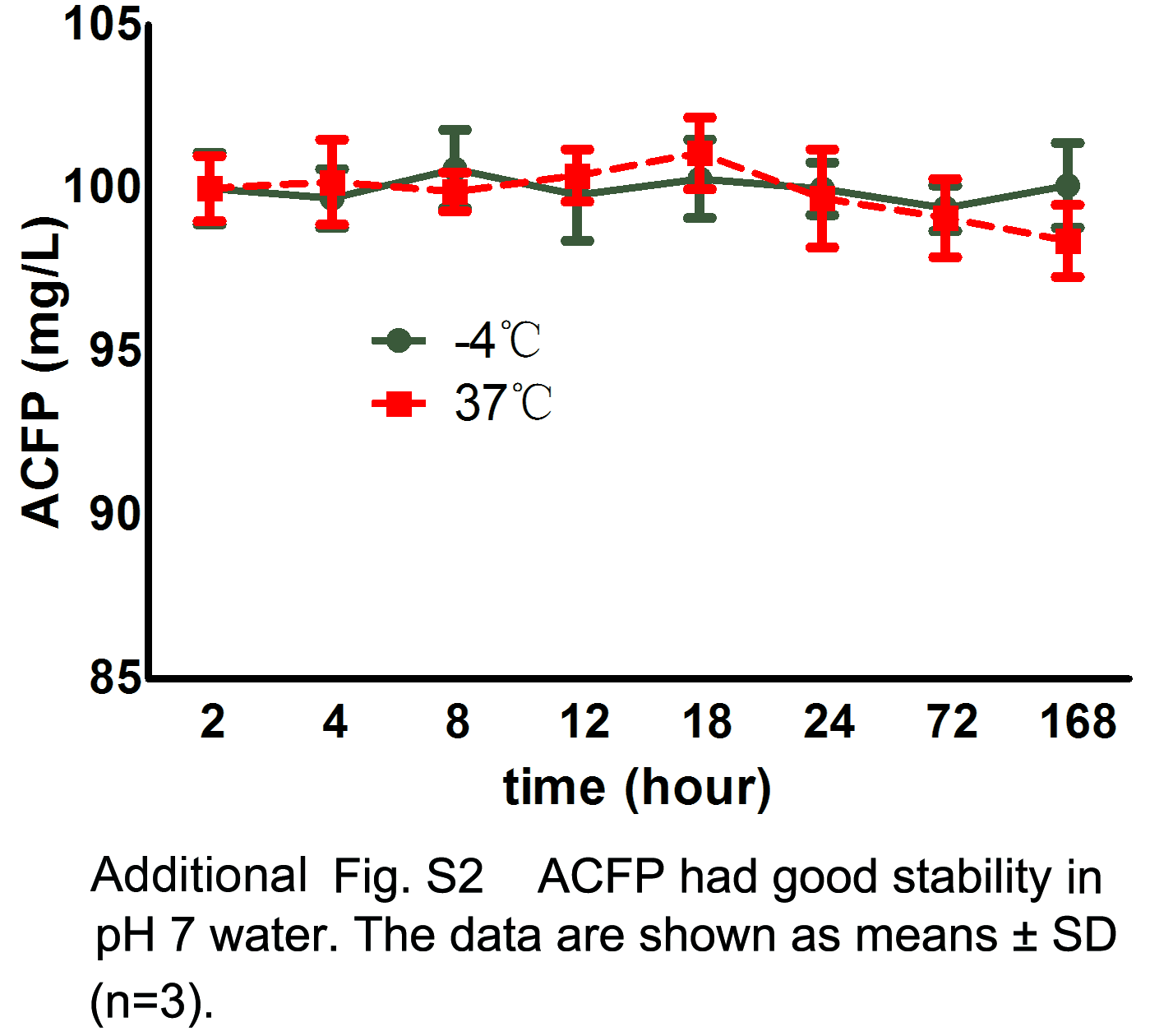

Supplement: Additional file 2: Figure S2. — ACFP had good stability in pH 7 water. The data are shown as means ± SD (n=3). (TIF 117 kb) [file 12885_2016_2281_MOESM2_ESM.tif]
